# Hematological Changes as Prognostic Indicators of Survival: Similarities Between Gottingen Minipigs, Humans, and Other Large Animal Models

**DOI:** 10.1371/journal.pone.0025210

**Published:** 2011-09-28

**Authors:** Maria Moroni, Eric Lombardini, Rudolph Salber, Mehdi Kazemzedeh, Vitaly Nagy, Cara Olsen, Mark H. Whitnall

**Affiliations:** 1 Radiation Countermeasures Program, Scientific Research Department, Armed Forces Radiobiology Research Institute, Uniformed Services University of the Health Sciences, Bethesda, Maryland, United States of America; 2 Veterinary Sciences Department, Armed Forces Radiobiology Research Institute, Uniformed Services University of the Health Sciences, Bethesda, Maryland, United States of America; 3 Radiation Sciences Department, Armed Forces Radiobiology Research Institute, Uniformed Services University of the Health Sciences, Bethesda, Maryland, United States of America; 4 Biostatistics Consulting Center, Armed Forces Radiobiology Research Institute, Uniformed Services University of the Health Sciences, Bethesda, Maryland, United States of America; Fundació Institut Germans Trias i Pujol; Universitat Autònoma de Barcelona CibeRES, Spain

## Abstract

**Background:**

The animal efficacy rule addressing development of drugs for selected disease categories has pointed out the need to develop alternative large animal models. Based on this rule, the pathophysiology of the disease in the animal model must be well characterized and must reflect that in humans. So far, manifestations of the acute radiation syndrome (ARS) have been extensively studied only in two large animal models, the non-human primate (NHP) and the canine. We are evaluating the suitability of the minipig as an additional large animal model for development of radiation countermeasures. We have previously shown that the Gottingen minipig manifests hematopoietic ARS phases and symptoms similar to those observed in canines, NHPs, and humans.

**Principal Findings:**

We establish here the LD50/30 dose (radiation dose at which 50% of the animals succumb within 30 days), and show that at this dose the time of nadir and the duration of cytopenia resemble those observed for NHP and canines, and mimic closely the kinetics of blood cell depletion and recovery in human patients with reversible hematopoietic damage (H3 category, METREPOL approach). No signs of GI damage in terms of diarrhea or shortening of villi were observed at doses up to 1.9 Gy. Platelet counts at days 10 and 14, number of days to reach critical platelet values, duration of thrombocytopenia, neutrophil stress response at 3 hours and count at 14 days, and CRP-to-platelet ratio were correlated with survival. The ratios between neutrophils, lymphocytes and platelets were significantly correlated with exposure to irradiation at different time intervals.

**Significance:**

As a non-rodent animal model, the minipig offers a useful alternative to NHP and canines, with attractive features including ARS resembling human ARS, cost, and regulatory acceptability. Use of the minipig may allow accelerated development of radiation countermeasures.

## Introduction

Use of nuclear energy for civilian and military application as well as exploitation of radiological materials for criminal intents pose risks to public health. The most recent example is the accident at the Fukushima power plant in Japan, which triggered a wide appreciation of the inadequacy of currently available countermeasures to face such emergencies. A full understanding of the pathophysiology of the acute radiation syndrome (ARS) is still incomplete; drug development is lagging behind. To facilitate approval of new drugs for selected conditions such as ARS, where human efficacy studies are neither ethical nor feasible, the FDA issued a draft guidance document (Animal Models - Essential Elements to Address Efficacy Under the Animal Rule) to allow consideration for marketing approval based on animal efficacy studies, provided the product is demonstrated safe in humans. Still, the shortage of suitable species for assessment of radiation exposure and understanding of radiation sickness is hampering research efforts, as the only well-characterized large animal models for ARS are non-human primates and canines. Swine provide a promising alternative to non-human primates and canines for advanced drug screening [1], and may represent a better model to study radiation damage to selected organs such as skin, kidneys and lungs [2]. Swine display a marked similarity in anatomy and physiology to human organs, and Gottingen minipigs are maintained with a controlled genetic and microbiological background. The cost is relatively low, and handling/husbandry and sampling/dosing procedures are straightforward.

We have previously shown that the irradiated Gottingen minipig is suitable for multiple large volume sampling for extended periods of time (60 days) following irradiation [3] and that, at doses bracketing the hematopoietic syndrome, it exhibits disease stages characteristic of ARS in humans and other large animal models. Moribund animals displayed signs of systemic inflammation and multi-organ dysfunction, including widespread internal hemorrhages and alteration in organ function reflected in blood chemistry; sepsis was present in a minority of the animals. The sequence of blood element declines followed the expected pattern, and bone marrow aplasia was confirmed by histological observations [4].

In the current study, we addressed more precisely the question of how the minipig compares to humans, NHP (non-human primates) and dogs in terms of hematological changes. Hematological changes (occurrence and duration of cytopenias, nadirs and onset of hematopoietic recovery) at the LD50/30 (radiation dose at which 50% of the animals succumb within 30 days) have been used in radiation biology for the past several years as a classic parameter of comparison among species; more recently, several diagnostic and prognostic indicators of ARS have been suggested in humans and NHP based on hematological values and serum biomarkers. Among these, the neutrophil-to-lymphocyte ratio and C-reactive protein have gained particular importance [5]. We determined the LD50/30 for the Gottingen minipig using 5 radiation doses (whole body gamma, 0.6 Gy/min) and 6 animals per dose, characterized the dynamics of platelet and neutrophil changes, and evaluated blood cell count- and CRP-based parameters as prognostic/diagnostic indicators of ARS in the Gottingen minipig. Our findings suggest that the pathophysiology of hematopoietic ARS in the Gottingen minipig is very similar to that observed in humans, NHPs and dogs. Although the LD50/30 in the minipig was lower and the survival curve steeper as compared to previous studies and to other models (4, 14–16), survival at specific doses has been quite reproducible over several years. Additional studies are underway to extend the survival curve to higher radiation doses, and to determine the responsiveness of the irradiated minipig to standard cytokine treatment.

## Materials and Methods

### Animal model and blood sampling

30 male Gottingen minipigs (4–5 months old, 9–11 kg) were purchased from Marshall Bioresources (North Rose, NY). Procedures were performed in accordance with protocols approved by the Armed Forces Radiobiology Research Institute (AFRRI) Institutional Animal Care and Use Committee. AFRRI is fully accredited by the Association for Assessment and Accreditation of Laboratory Animal Care, International. The protocol was approved by the Armed Forces Radiobiology Research Institute IACUC Committee. Animals received appropriate environmental enrichment, and all efforts were made to minimize suffering [3]. The first blood sample was taken 6 days prior to VAP implantation via the cranial vena cava. The day of irradiation (3 weeks after VAP surgery) was considered day 0. Additional blood samples were collected on days −14, −7 and −1, at +3 h, +7 h, +11 h, +27 h, +31 h and +35 h, and on days +2, +3, +7, +10, +14, +17, +20, +24, +27, +30 and, for selected cases, +60, depending upon survival.

### Blood counts

Blood was collected in EDTA tubes and immediately processed for complete blood counts with differentials (CBC/diffs) using the ADVIA 2120 (Siemens Medical Solutions Diagnostics, Ireland). The remaining plasma was aliquoted and stored at −80°C for further analysis. At the end of the study, animals were anesthetized with tiletamine/zolazepam (Telazol®, 6–8 mg/kg IM) and humanely euthanized with an intravenous overdose of sodium pentobarbital (Euthasol®), according to current American Veterinary Medical Association (AVMA) guidelines. A full necropsy was performed in all cases and histopathological examination (Hematoxylin and Eosin (H&E)) of all tissues was conducted.

### Irradiation procedure and dosimetry

Prior to irradiation, animals were anesthetized with tiletamine/zolazepam (Telazol®, 6–8 mg/kg IM) and administered a bolus injection of atropine (0.05 mg/kg IM) to decrease salivary secretions. Minipigs were irradiated bilaterally in a sling in the AFRRI Co-60 facility with 1.6, 1.7, 1.8, 1.9 or 2 Gy, at the dose rate 0.5–0.6 Gy/min, as described elsewhere [4].

### C-reactive protein

Concentrations of C-reactive protein (CRP) in blood plasma samples were assessed using a commercially-available porcine-specific ELISA kit (Innovative Research, MI, USA), according to the manufacturer's instructions. Detection limits were 6.25–200 ng/mL. Single-use plasma aliquots stored at −80°C were thawed on ice, diluted 1∶2000 and measured in duplicate. Samples were measured using a plate reader (SpectraMax M5, Molecular Devices, CA, USA) set at an absorbance of 450 nm.

### Statistical analysis

Basic descriptive analysis and two-sided Student's t-tests were done in Excel. Spearman Rank correlation was calculated using the Free Statistics and Forecasting Software Calculator at: http://www.wessa.net/rankcorr.wasp. Probit regression analysis was used to generate the probit curve, using the PASW Statistic 18 software, SPSS Inc, IL, USA. Survival curves were plotted using the Kaplan-Meier method.

## Results and Discussion

### Hematological and histological changes at the LD50/30

#### Comparison between Gottingen minipigs, humans and other large animal models

We are studying the effect of acute radiation in the Gottingen minipig to evaluate whether this model is suitable for rapid development of advanced countermeasures, as a complement to the existing large animal models for the ARS, the NHPs and canines. Comparison of lethal responses in different species under various conditions of irradiation has been achieved, over the years, through evaluation of hematological values at the hematopoietic LD50 dose, the radiation dose that results in 50% of lethality (LD50).

**Figure 1:**
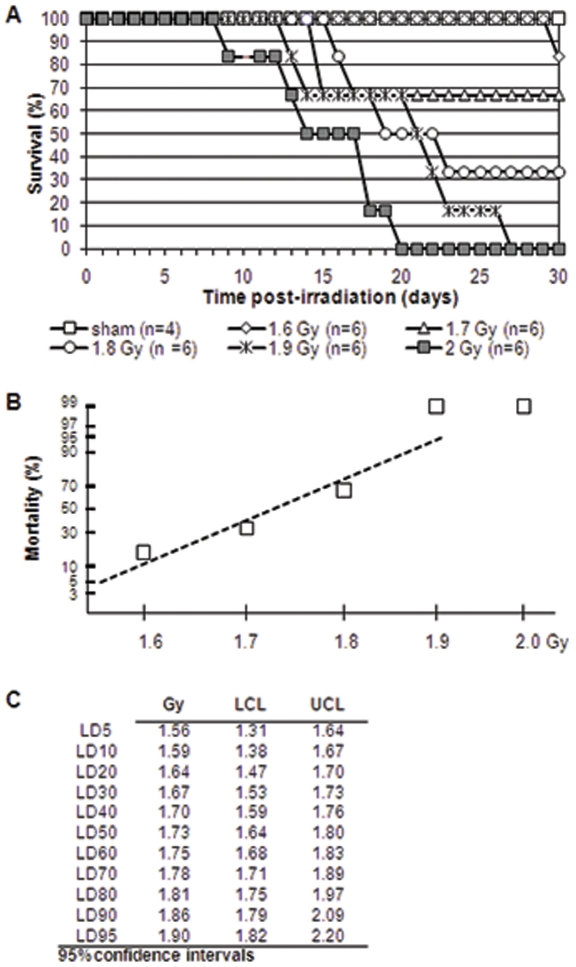
Dose response curves for bilateral gamma-irradiation of Gottingen minipig. Six animals per dose were irradiated with Cobalt-60 (0.6 Gy/min). Survival was followed for 30 days after exposure and data were plotted as Kaplan-Meier curves (Panel A). Probit analysis was used to fit survival curves and calculate dose-response (Panel B) and confidence intervals (Panel C).

We established the LD50/30 in the Gottingen minipig using 5 doses (total body irradiation, TBI, bilateral, Co-60, 0.6 Gy/min) and 6 animals per dose, and monitored hematological changes over 30–60 days. All animals either succumbed by 30 days, or were showing clear signs of hematological recovery before 30 days (see below). Baseline hematology data were obtained from 4 sham-irradiated animals, subjected to the same treatment and sampling schedule as the irradiated ones. From our study, the LD50/30 for the Gottingen minipig was 1.73 Gy, (Cobalt-60, 0.6 Gy/min, TBI, bilateral) (Figure 1), in agreement with the 1.7–3.7 Gy range for LD50/30s previously reported for swine, including domestic swine and minipigs [6]–[8]. This value corresponds to about half the value of humans (accepted estimated value 3–4 Gy with no supportive care) [9], while the NHP are twice as radioresistant as humans. The relative radiosensitivity of the Gottingen minipig raises questions concerning the possibility of genetic mutations, defects in DNA repair, the effects of inbreeding, etc. In terms of mutations, the proportional dwarfism characteristic of the Gottingen minipig, referred to as “pituitary dwarfism”, is found in a number of farm animal breeds, i.e. Dexter cattle, Shetland pony and, unlike the “achondroplasia” type of dwarfism, is a genetically fixed trait and not considered a genetic defect [10 Simianer]. Deficiency in DNA repair mechanisms does not seem to explain the radiosensitivity in the Gottingen minipig, since DNA double-strand break repair kinetics in blood cells, based on gammaH2AX focus expression, have been found to be similar to that of in humans and NHPs (C. Redon, personal communication). Inbreeding for this strain is controlled and kept under 10% [10]–[11]. Mapping of the minipig genome is ongoing [12], and may provide some insight into this issue which for the moment remains unresolved.

**Figure 2:**
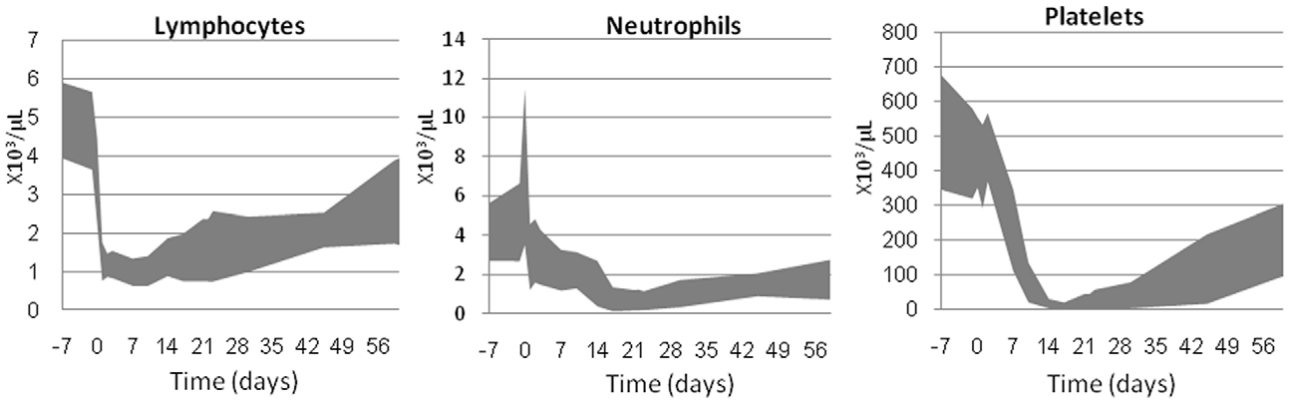
Ranges (min, max) of blood cell loss and recovery in irradiated minipigs. Graphs represent the minimum and maximum blood cell values observed over time from 30 animals irradiated with doses ranging from 1.6 to 2.0 Gy at 0.1 Gy dose intervals and pooled together (6 animals/dose).

Although the LD50/30 was significantly lower than that of humans, hematological changes in the minipig (Figure 2) were very similar to what has been observed in victims of criticality accidents. We considered the METREPOL hematology category H3 (autologous recovery certain with high risk critical phase) [13] as a reasonable representation of the pathophysiology of humans at the LD50/30. In patients with the H3 level of hematopoietic effect, the hematopoietic depression resulted in rapid loss of lymphocytes, reaching minimum values within 2 days. Disappearance of granulocytes from peripheral blood started to occur within 2–4 days, and was preceded by an initial granulocytosis during the first two days after exposure, due to mobilization from marrow and possibly spleen storage pools. An abortive rise could be noted starting around day 5 and neutropenia (<0.5×10^3^ cells/µl) was reached around days 10–15. The platelet decline was steady and relatively slow; thrombocytopenia (<20×10^3^cells/µl) was reached by 2–3 weeks. Nadirs for neutrophils and platelets were reached around 3–4 weeks; the duration of cytopenias was approximately 20 days. Recovery began 5–6 weeks post-exposure with platelets returning to normal values after approximately 8 weeks [13]. Very similar dynamics were observed for the Gottingen minipig (Figure 2), with a rapid decline in lymphocyte counts within 48 hours, an initial stress response of granulocytes followed by a decline and abortive rise before reaching neutropenic levels by 14–17 days, and slow loss of platelets following an initial shoulder, with thrombocytopenic levels reached by 10–14 days. At doses close to the LD50/30 (Table 1), nadirs for platelets and neutrophils were reached around days 14–17 and 14–23, respectively. The duration for both thrombocytopenia and neutropenia was around 1–2 weeks, and recovery started around 3–4 weeks after exposure (Table 2). Full recovery of platelets required more than 8 weeks. Only eight animals out of 30 showed signs of anemia (hematocrit range 6%–13%; median 11.6%). Anemia is not considered a survival-limiting factor in humans, but increases in importance in NHP, dogs and guinea-pigs and is most important in mice and rats [14].

**Table 1:** Platelet and neutrophil nadirs and duration of cytopenias of irradiated minipigs.

	Survival		Thrombocytopenia (plt<20/nL)			Neutropenia (ANC<0.5/nL)			% cytopenic
Dose	%	median	nadir		duration	nadir		duration	animals
(Gy)		day	day	plt/nL	weeks	day	ANC/nL	weeks	
**1.6**	0.8	30	17	4.5	>1	20	0.21	∼1	83%
		(29–30)	(14–23)	(2–10)		(17–20)	(0.12–0.39)		
**1.7**	0.7	30	14	3.5	>1–2	17	0.16	>1	100%
		(14–30)	(14–17)	(1–17)		(14–23)	(0.09–0.46)		
**1.8**	0.3	21	14	2.5	>2	17	0.13	>2	100%
		(16–30)	(14–20)	(1–9)		(17–17)	(0.03–0.31)		
**1.9**	0.0	21.5	14	2.5	no recovery	17	0.04	no recovery	100%
		(14–27)	(14–17)	(1–4)		(14–20)	(0.03–0.17)		
**2.0**	0.0	18	14	3	no recovery	15.5	0.07	no recovery	100%
		(14–20)	(14–17)	(1–9)		(14–20)	(0.04–0.28)		

Range values (min, max) are reported in parenthesis; plt: platelets; ANC: absolute neutrophil number.

**Table 2:** Compiled published data for hematological dynamics in human, minipig, NHP and canine after irradiation.

	Dose			Thrombocytopenia				Neutropenia			
	Gy	Rate	Type	Nadir	Duration	Recovery (onset)	Recovery (Full)	Nadir	Duration	Recovery (onset)	Recovery (Full)
**Human**	H3 (hematopoietic recovery possible)			3–4 weeks	2–3 weeks	>5 weeks after exposure	∼2 mo	2–3 weeks	2–3 weeks	4–5 weeks after exposure	∼6–8 weeks
**Minipig – Gottingen**	2	0.6 Gy/min	Co-60 Gamma	16 d	∼2 weeks	∼3–4 weeks after exposure	>2 mo	18 d	1–2 weeks	∼3–4 weeks after exposure	>2 mo
**NHP – Rhesus**	5	<50–500 msec pulse	TRIGA reactor (1∶1 neutron gamma)	13 d	10–12 d	∼15 d	32 d	∼11–13 d	16.5	∼17 d	∼24 d
	7	0.4 Gy/min	Co-60 Gamma	12 d	12	16 d		15 d	14.8	18 d	∼20 d
	6	13 cGy/min	x-ray		∼7 d		∼18 d		∼19		∼24 d
	7	0.2–0.4 Gy/min	Co-60 Gamma	15 d	12–20 d	∼16–18 d	>40 d	6 d	∼15	∼17 d	24 d
**Baboon**	5	5.5 Gy/∼3 min	Pulsed reactor (Neutron: gamma = 5.5)	13 d			25 d				
**Canine - Beagles & others**	2–4	0.04–.0.1 Gy/min	X-Ray	∼20 d			>35–45 d	∼14–17 d	∼17 d	∼17–20 d	∼35–45
	2	0.065 Gy/min		12 d	∼10 d	∼22 d	∼38 d	∼18 d		∼22 d	45

REFERENCES: Human data from: ref [33]. Swine data from: ref [4]. NHP data from: refs [19], [34]–[40]. Canine data from: refs [17]–[18], [41]–[42].

For the comparison of hematological dynamics in the irradiated minipig with other large animal models (Table 2), we referred to published studies that used radiation doses close to the accepted LD50/30 values for NHP (6.5 Gy) [14], [15] and dogs (2.6 Gy) [16]. In the Beagle dog irradiated with 3 Gy (Co-60, 10 cGy/min, TBI), nadirs were reached around days 14–17 (neutrophils) and 20 (platelets) [17], [18]. Neutropenia and thrombocytopenia lasted for about 2 to 3 weeks. For Rhesus monkeys irradiated with 7 Gy, nadirs for platelet and neutrophil counts were reached around 12–15 days and recovery started 2–3 days later. Neutrophil and platelet counts returned to almost normal values after approximately 2–3 weeks [19], [20]. These values are very close to those obtained in the Gottingen minipig.

The similarities in rate of hematopoietic changes between swine and humans, consistent with NHP and canines, support the suitability of the minipig as a model for the hematopoietic syndrome of ARS. During steady-state production, the mean value for myelocyte-to-blood transit time is 8–10 days for human [13], 6–7 days in swine, and 4–6 days for dogs [21]. Neutrophil half lives in circulation are comparable in all these species, about 6.6 hours for man, 8 hours for swine [22], and 7 hours for dogs [23]. As for platelets, total transit time from the appearance of the most immature megakaryocyte in the marrow to the release of platelets in the human peripheral blood is 8–10 days, and the life span is 8–10 days [13]; total megakaryocyte maturation time in pigs ranges from about 5 to 10 days and life span of platelets is 5–7 days [24]; in dogs, the platelet life span is 9 days [25]; and in Rhesus monkey the life span is about 6.5 days [26]. The lifespan of RBC is around 80–100 days in the swine [27], 100–115 in the dog [27], 85 days in Rhesus monkey [28] and 120 days in humans [13].

**Figure 3:**
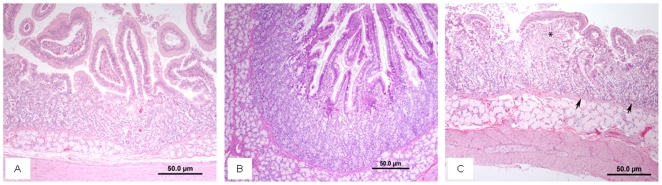
Histological examination of crypts and villi in the duodenum of irradiated minipigs (1.6 to 2.0 Gy). Samples were collected and fixed at necropsy. Panel A (1.6 Gy, H&E 100×). Villar length is consistent throughout the section, and is of normal length, lined by a single layer of columnar epithelium with basilar nuclei and abundant eosinophilic apical cytoplasm. Underlying crypts are densely packed. Panel B (1.9 Gy, H&E 100×). Villar length remains consisted in length and in morphology of the overlying epithelium as well as the abundance and morphology of the underlying villar crypts. Panel C (2.0 Gy, H&E 200×). Multifocally throughout the section, villi are blunted and fused (asterix) with multiple villi recovering by denudation by being covered by a single layer of columnar epithelium. Underlying crypts (arrows) have piled epithelium and occasionally are tortuous; however there is no apparent loss of crypts.

Unlike dogs, where diarrhea, bloody feces, and ulceration in the cecum/colon/rectum occurred already at the LD50/30 [16], in the Gottingen minipig widespread hemorrhages were present, but no signs of diarrhea or necrosis/ulceration were observed up to 1.9 Gy. Histopathological examination indicated that in duodenum, jejunum and ileum, villar length, epithelial morphology, crypt numbers and morphology were within normal limits through radiation doses of 1.9 Gy (Figure 3a and Figure 3b). Animals dosed at 2.0 Gy began to show signs of regeneration after denudation of the villi, presumably secondary to radiation injury. This consisted primarily of blunting and fusion in which multiple denuded villi fused together and a single regenerative layer of epithelium stretched to cover all affected villi (Figure 3c). Crypts were consistent in overall number with those in animals undergoing lesser radiation doses; however, the crypt epithelium underwent mild morphological changes comprising increased piling of the epithelial cells and occasional crypt dysplasia comprised of elongated tortuous and branching crypts, especially subjacent to the blunted and fused villi. Histopathological changes in the large intestine consisted of varying degrees of mucosal and submucosal hemorrhage with no radiation-associated alterations of the mucosal epithelium.

#### Prognostic and diagnostic indicators

To further assess similarities between the Gottingen minipig and humans and accepted large animal species, we measured ARS diagnostic and prognostic indicators related to the dynamics of blood element declines. In irradiated humans [29], platelet counts and number of days to reach critical values for granulocytes and thrombocytes were found associated with ARS grades [30]. We previously showed that in the Gottingen minipig, platelet cut-off values could be used to predict mortality [4]; in the current study (30 animals), we measured the Spearman rank correlation coefficient (ρ) between 30 day survival (or less than 30 day survival) and (i) absolute platelet counts (at days 10, when the prodromal phase starts to taper off, and 14, when the first deaths occur); (ii) number of days to reach critical platelet values i.e. minimum normal levels (300,000/µL), thrombocytopenic levels (<20,000/µL) and nadir; (iii) number of days of thrombocytopenia until the first death (day 14). All these parameters were found significantly associated with survival (Table 3). Also, (iv) neutrophil counts at 14 days as well as (v) neutrophil relative increases over pre-irradiation values at 3 hours after irradiation were significantly associated with survival (Table 4), again reinforcing the similarities in pathophysiology of the ARS between swine and human [30], [31]. In agreement with the NHP data of Stickney et al [20], we found that in the Gottingen minipig the cumulative number of days of thrombocytopenia (r_s_ −0.66) but not (vi) neutropenia (r_s_ −0.19) was significantly associated with outcome. Recently, we have shown that in minipigs C-reactive protein (CRP), an acute phase protein found increased in irradiated victims and proposed as a biomarker of irradiation [5], significantly increased after irradiation, and that at ≥14 days post-radiation elevated CRP levels were correlated with prognosis [4]. Here, we measured the ratio between CRP and platelet counts (vii); a highly significant negative association with survival was found starting at day 10 (r_s_ = −.65, p≤0.001), more so than CRP values alone; a statistically significant difference between 14 day CRP levels in surviving versus non-surviving animals was confirmed in the current study (p<0.05).

**Table 3:** Spearman rank correlation (ρ) of platelet counts-based parameters with survival in irradiated G. minipig (n = 30).

	Counts/nL	Counts/nL	Counts/nL	Days to reach	Days to reach	Days to reach	No. of days with
Survived	at 7 day	at 10 day	at 14 day	300 plt/nL	20 plt/nL	nadir	<20 plt/nL
median	304	53.5	6	7	12	17	1.5
min	110	20	3	4	10	14	0
max	344	134	30	8	17	23	4
**Not-survived**							
median	188	18	3	5	9.5	14	4
min	119	3	1	3	9	10	2
max	278	55	7	6	11	17	4
**ρ**	0.49	0.627	0.64	0.6	0.62	0.61	−0.66
**p value**	0.049	0.0006	0.0002	0.001	0.0008	0.0009	0.0001

Plt: platelets; p value (Student's T-test).

**Table 4:** Spearman rank correlation (ρ) of ANC counts- and CRP-based parameters with survival in irradiated G. minipig (n = 30).

Survived	ANC Counts/µL	ANC relative increase	No. of days	CRP:plt	
	at 14 day	at 3 hr	<0.5 ANC/µL	10 day	14 day
median	0.9	1.58	0	0.3	2.6
min	0.4	1	0	0.1	0.4
max	2.7	3.4	1	1.1	7.1
**Not-survived**					
median	0.335	2.75	0	1.2	9.7
min	0	1.2	0	0.3	2
max	1.1	10	3	8.5	68
**ρ**	0.569	–0.58	–0.19	–0.65	–0.61
**p value**	0.002	0.001		<0.001	0.001

ANC: absolute neutrophil counts; CRP: C-reactive protein; CRP:plt : CRP-to-platelets ratio; p value (Students' T-test).

**Figure 4:**
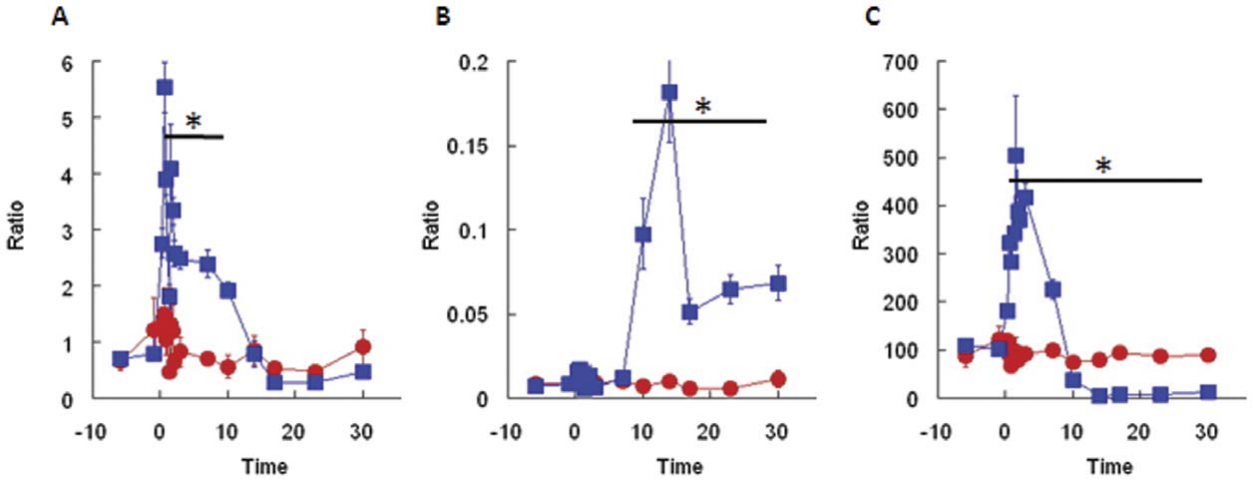
Ratio of hematological parameters. Neutrophil-to-lymphocyte (A), neutrophil-to-platelet (B), and platelet-to-lymphocyte (C) ratios were calculated for sham-irradiated animals (diamonds, n = 4) and for animals irradiated with 1.6–2.0 Gy (squares, n = 30). Ratios were calculated for each individual animal and results were pooled together. Asterisks (*) indicate statistically significant difference between groups (Student T-test); bars represent standard errors.

In terms of diagnostic indicators, rapid assessment of exposure is important to provide the best medical care and optimize the use of resources. In addition to the abundance of proposed indicators of exposure, hematological responses can provide simple and reliable early indicators. Lymphocyte drops and hematological changes over the first few days after exposure have been used for dose estimates and for criticality accidents [29], [32]. In the minipig a rapid decline of lymphocyte counts was observed over the first 12 hours, which is indicative of severe exposure to radiation according to Goans and colleagues (op. cit.). Accordingly, the neutrophil-to-lymphocyte ratio, a recently developed diagnostic indicator considered more practical than lymphocyte count alone in humans and NHPs [5], clearly separated irradiated versus non-irradiated animals at 3 hours and up to 10 days after exposure (Figure 4). Along the same line, we determined the neutrophil-to-platelet and the platelet-to-lymphocyte ratios in un-irradiated versus irradiated animals. Both those ratios were significantly different between the two groups up to 30 days after exposure; for the platelet-to-lymphocyte ratio, the extent of the difference was about 100-fold larger than observed for the neutrophil-to-lymphocyte ratio, and covered a time window from 3 hours to 30 days (Figure 4), thus making this index more sensitive than the neutrophil-to-lymphocyte ratio.

We conclude that the pathophysiology of ARS in the Gottingen minipig is similar to that observed in humans, NHP, and dogs in terms of hematological dynamics and importance of thrombocyte counts. The Gottingen minipig appears to be a suitable alternative large animal model to study the radiation-induced hematopoietic syndrome and to test radiation countermeasures. Additional experiments to characterize other aspects of ARS in Gottingen minipigs are ongoing.
